# Biochemical Changes and Biological Origin of Key Odor Compound Generations in Pig Slurry during Indoor Storage Periods: A Pyrosequencing Approach

**DOI:** 10.1155/2018/3503658

**Published:** 2018-09-12

**Authors:** Yu Na Jang, Min Woong Jung

**Affiliations:** National Institute of Animal Science, Rural Development Administration, Wanju-gun, Jeollabuk-do, Republic of Korea

## Abstract

Production of odors is a complex process. Many bacterial species are involved in the production of an extensive array of key odor compounds in stored pig slurry. Understanding of basic microbial communities and their role during storage periods is an essential way to control and prevent the odors generations. In this aspect, the pig slurry samples were taken directly from deep pits of finisher pig building every two weeks, their biochemical changes were analysed, and the indigenous bacterial communities that involve in offensive odor producing compounds were identified. The SCFA, BCFA, phenols, and indoles levels altered drastically in the slurry during storage periods. The COD, BOD, SS, P_2_O_5_, TKN, and NH_4_-N were increased in the stored slurry. Bacterial ecology indicates* Firmicutes* and* Bacteroidetes *phyla were dominantly found in pig slurry. Odorants produced in pig slurry were correlated with bacterial communities. Phenols, indoles, SCFA, and BCFA productions were positively correlated with bacteria species which comes under phyla of* Firmicutes *and* Bacteroidetes. *It seems that bacterial species under* Firmicutes* and* Bacteroidetes *phyla play an important role in the offensive odor compounds production. Taken together, the prevention of these phyla bacterial growth and early discharge of pig slurry might reduce the offensive odor production.

## 1. Introduction

An emission of odorants has attracted massive consideration due to their harmful effects to human health and atmospheric environment [1, 2]. Environmental pollution from animal manure is a critical issue and is an acute and serious in countries with high level of animal's production on a limited land base for manure disposal. In Korea, the amount of pig slurry was continuously increased from 4, 370 million tons in 2009 to 4,724 million tons in 2013[3]. More than 54% of civil complaints about malodor were accounted from swine farming house in Korea[4]. In Korea, two different pig houses have been used for swing farming (open ventilation system and slurry storage system). It is a main reason for the odor emissions [4]. For that Korean government has announced an offensive odor control law and provided information about where livestock buildings are constructed [5, 6]. Pig slurry generally is stored in deep pit under the pig building for a couple of weeks to months before cleaned out. Slurry composition is based on not only well factors such as diet and slurry management [7, 8] but also age of slurry. Degradable and nondegradable volatile solids produce the organic matter (OM) in slurry. During this condition, most of organic matters degradable by microbes cause increases in fibrous content in the slurry [9]. Further, bacterial degradation of slurry accumulates fermentative metabolic products such VFAs and mineralization products of nitrogen such as NH_3_ and N_2_O [7]. More than 200 compounds are known to be associated with livestock odor, among these; several compounds are contributed to the offensive odor [10, 11]. Particularly, amines, ammonia, volatile fatty acids, phenols, and indoles are the key components related to offensive odor in feedlot manure [11–14]. Identification of biochemical changes and biological origins of offensive odor compounds production in pig slurry is a first step to develop the new strategy for controlling key components related to malodor production during storage periods in deep pits. Number of studies deals with microbial population in pig faces [15–17]. However, few of the studies only reported the causes of odor and its correlation between the bacterial communities and odor compound productions [18, 19]. The aim of the current study is to analyse the changes in odor compounds and microbial communities in pig slurry and investigate their correlation between key odor components and bacterial communities in stored pig slurry.

## 2. Material and Methods

### 2.1. Sample Collection

Pig slurry during fattening periods (6-96 days) carried out with 85-98 pigs (77 days old with initial weight 25±2.50kg) was collected from deep pits every two weeks (17 m^3^× 14 pits = 238 m^3^) in the month and season between July 11 and October 10 and summer and autumn, respectively, at National Institute of Animal Science. The animals were fed with basal diet formulated according to the Korean Feeding Standard [20]. The animals were housed in whole slatted pens (7 rooms). During the fattening periods, the slurry in the deep pits was mixed in order to avoid stratifications and then samples were taken once a week for 96 days at the same location in the center of the slurry pits. Samples were collected at the same location for every time, stored in an icebox, and then moved to the laboratory. Then the samples were separated and stored at 4°C and −20°C for physiochemical and odorous substance analysis, respectively. For the analysis of volatile organic compounds, VFAs, phenols, and indoles were sampled at 0.1L/min for 5 min using SIBATA pump (MP-∑30KN||). For analysis of sulfur compound, complex odorants were sampled using a vacuum type lung sampler equipped with a Tedlar bag which was used as an indirect sampling method. Sampling was performed within 3 minutes as soon as possible to monitor the generation of odor. The flow was maintained using SKC pump (224-PCXR8) at 4L/min for 3 min (US EPA air method).

### 2.2. Physiochemical Characterization of Pig Slurry Samples

The pH of the slurry was measured directly with a glass electrode pH meter. The moisture content, total organic matters, biological oxygen demand (BOD), chemical oxygen demand (COD), total ammonia (NH_4_), and total Kjeldahl nitrogen (TKN) were analysed by steam distillation coupled with a titration unit; phosphorous pentoxide (P_2_O_5)_ and nitrate nitrogen NO_3_-N were analysed according to the standard protocol. The physicochemical analysis of samples was repeated three times through three different sampling processes.

### 2.3. Volatile Fatty Acids

Ten milliliters of pig slurry was mixed with 2 ml of 25% metaphosphoric acid and 0.1ml saturated mercury chloride solutions in 15 mL plastic tubes, centrifuged at 3134 g for 20 min at 20°C and collected supernatant. Further, supernatant was centrifuged at 12000g for 10 min and filtered through a 0.2 *μ*m filter. The filtrates were used for VFAs analysis. The concentrations of VFAs were analysed according to the standard protocol using gas chromatography (6890N, Agilent, SantaClara, CA, USA), equipped with DB-FFAP column (30 m × 0.25 mm × 0.25 *μ*m; Agilent, Santa Clara, CA, USA) and flame ionization detector (FID). Briefly, 0.5 *μ*L with 10:1 split ratio of sample was injected. The initial temperature of the oven was 60°C for 2 min; further temperature was increased to 180 at 10°C/ min. The injection and detection ports were maintained at 250°C [21]. The volatile fatty acids analysis of samples was repeated three times through three different sampling processes.

### 2.4. Phenols and Indoles

The slurry samples were collected from deep pits once a week and centrifuged at 3134 g for 20 min at 20°C and then equal volume (4mL) of supernatant and chloroform was mixed. 60 *μ*L of 4 M NaOH was added to the mixture in a 20 mL glass vial. Further, this mixture was centrifuged at 3134 g for 20 min at 20°C. The chloroform layer was separated and transferred to new vials. The phenols and indoles were analysed according to the standard protocol using GC (7890B, Agilent, Santa Clara, CA, USA), equipped with a DB-1MS column (60 m × 0.32 mm × 0.25 *μ*m; Agilent, SantaClara, CA, USA) and mass spectrometer detector (MSD). The sample injection was 2.0 *μ*L with a 5:1 spilt ratio. The initial temperature of oven was 40°C for 5 min; furthermore, temperature was increased to 230 at 10°C/ min. This 230°C was maintained for minutes. The injection and detection ports were maintained at 250° [21]. The phenols and indoles analysis of samples were repeated three times through three different sampling processes.

### 2.5. Detection of Odor Compounds in Atmospheric Air

The volatile organic compounds (VOCs), VFAs, phenols, and indoles were sampled at 0.1L/min for 5 min using the 3-bed tube (Carbopack C: Carbopack B: Carbopack X, 1:1:1). The analysis system utilized a system in which thermal desorption (TD) and GC/MSD are connection system. The TD (unity + air server, Markes, UK) cold trap condition increases low temp 5°C to high temp 300°C, split flow was 10:1, and flow path temperature was maintained at 150°C. VOCs, VFAs, phenols, and indoles were analysed using a GC (6890N/5973N, Agilent, Santa Clara, CA, USA) equipped with a CP-Wax52CB column (60m × 0.25mm × 0.25um) and a MSD. The oven temperature was initially 45°C for 5 min, increasing to 250°C at 5°C/min, which was then held at 250°C for 4 min. The ion source temperature was maintained at 230°C.

The sulfur compound was sampled using the aluminum Tedlar bag. TD and flame photometric detector (FPD) were used. The column was equipped with CP-Sil 5CB (60m × 0.32mm × 5 um). The initial oven temperature was 80°C for 5 min, increasing to 200°C at 8°C/min and then holds time to 5 min. The odor compounds analysis of samples was repeated three times through three different sampling processes.

### 2.6. Bacteria Ecology by Pyrosequences Method

According to the protocol of Fast DNA Spin Kit (MP Bio, Santa Ana, CA, USA) the total genomic DNA was extracted from slurry at every week. The humic acid interference was removed by power clean DNA cleanup Kit (MP Bio, Santa Ana, CA, USA). The extracted gDNA was amplified using primers targeting the V1-V3 hypervariable regions of the bacterial 16s rRNA genes (27F-5′-adaptor 2-AC-GAG TTT GAT CMT GGC TCA G-3, 518R-5′-adaptor1-AC-X-WTT ACC GCG GCT GCT GG-3′ indicate unique 7 to 11 barcode sequences inserted between the 454 life Sciences adaptor A sequences and common linker AC). The PCR amplification conditions were sued as initial denaturation at 95°C for 5 min, followed by 25 cycles of denaturation at 95°C, annealing at 55°C for the 30s, elongation at 72°C for 30s, and final extension at 72°C for 7 min. The PCR products of each sample were subjected to the pyrosequencing analysis (Chun Lab, Seoul, Korea) by 454 GS FLX Titanium sequencing system (Roche, Pleasanton, CA, USA).

#### 2.6.1. Preprocessing of Data Sets

The sequencing reads from each sample were separated by unique barcodes. Then, barcode, linker, and PCR primer sequences at both sides were removed from the native reads. The final sequences were subjected to filtering process included only reads containing >300bp and an average quality score was > 25.

#### 2.6.2. Taxonomic Assignment of Individual Sequencing Reads

High-quality bacterial reads are used for taxonomic assessment using an EzTaxon-2 database and robust global pair wise sequencing alignment, coupled with the BLAST search tool (). The sequences could be matched with an EzTaxon-2 database at the species level (97%) which were subjected to check the chimeric sequences using the UCHIME program (Edgar et al., 2011). The operational taxonomic units were generated using the CD-HIT program at a 97% similarity level. The Shannon Weaver diversity index, Chao 1 richness index, and goods library coverage were calculated by the Mothur package [22].

### 2.7. Statistical Analysis

The data which are generated from the experiments were subjected to analysis of variance for a completely randomized design using the linear model procedure of SAS software and multiple statistical comparisons between odour compounds and bacterial community with p<0.05 level significance [23]. Hierarchical clustering, principal component analysis (PCA), and principal coordinate analysis (PCoA) were performed. Pearson's correlation coefficient was used to determine the linker between bacterial genera and odor compounds.

## 3. Results

Table 1 shows the detection of environmental temperature and atmospheric humidity during different storage periods. The environmental temperature and humidity conditions have fluctuated at every week. The average minimum temperature and humidity of environmental were 28°C and 81%, respectively. The average maximum temperature and humidity of environmental at every week were 29°C and 89%, respectively

### 3.1. Effect of Storage Periods on Odor Compounds in Deep Pit Slurry

Table 2 compares the key odor compounds productions from stored pig slurry in deep pits during different storage periods. The highest short-chain fatty acids (SCFA) such as acetic acid, propionic acid, butyric acid, and valeric acid concentrations were found in stored pig slurry (8132.5 mg/L) at 4th week as compared to first week of slurry (2234.2 mg/L). Later, this concentration was reduced to 1934.1 at 14 week. The total branch chain fatty acids (BCFA) such as isobutyric acid and isovaleric acid tended to increase from 96.1 to 667.9 mg/L (693.96%) at 6th week. Furthermore, BCFA levels were decreased to 127.3mg/L at 14th week. The total phenols including phenol (PhA1) and p-Cresol (P-C) concentrations were highest at the 6th week as compared to previous weeks. After that this concentration was reduced at 14th week. The total indoles such as indole (ID) and skatole (SK) were higher at 9th week of storage. Subsequently, concentrations of indoles were continuously reduced in stored pig slurry.

### 3.2. Physiochemical Characterization of Stored Slurry

Further, we analysed concentration of pollutants related components in the pig slurry collected from deep pits at different weeks (Table 3). The concentration of pollutant markers such as BOD, COD, and SS steadily increased from 37020, 13600, and 13400 to 41460, 19200, and 37200, respectively, up to 10 weeks. However, the maximum concentration of BOD, COD, and SS is noted at weeks 5 and 6, respectively. The organic P_2_O_5_ was increased from 0.1 to 0.44% at week 5. The pH of the slurry increased from 5.8 to 6.0 at week 11. The moisture content of slurry was slightly reduced at weeks 4 and 5, but after the 5th week, the moisture content of the slurry was increased to 97.3 at week 14. Maximum total nitrogen (TKN) and ammonia (NH4-N) concentration were noted at 5th and 4th week, respectively. Then, the concentration of TKN and NH4-N was reduced gradually at 14 weeks.

### 3.3. Atmospheric Mixed Odor Ratio during Storage Periods

**Figure 1:**
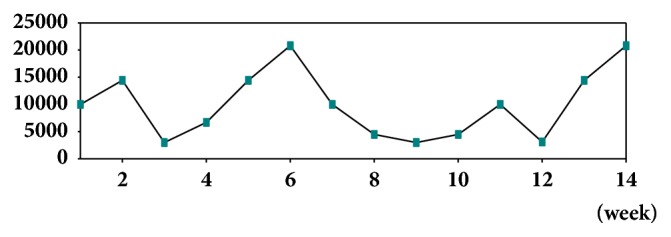
The ratio of mixed odors generated by the pig slurry during storage periods.

The atmospheric odor ratio (AOR) produced by slurry was investigated at different weeks and their results were presented in Figure 1. The results showed that AOR ratio was up and down in a time-dependent manner. The AOR ratio was higher (20800) at weeks 2, 6, and 14 than the other storage weeks.

### 3.4. Atmospheric Odorant Concentration Generated by Pig Slurry

Key components associated with atmospheric odorant were analysed and the details were given in Table 4. The major contributors to the atmospheric odorant were sulfur compounds, VOCs, VFAs, phenols, and indoles. The sulfur compounds such as hydrogen sulfide (H_2_S) and methyl mercaptan (MM) were increased continuously from 62.5 and 186 at week 1 to 1685 and 249 at weeks 11 and 10, respectively. The volatile organic compounds MEK, isobutyl alcohol, and styrene were higher at week 14; the concentration of toluene, n-butyl acetate, and ethylbenzene was increased until week 10. Most of VFAs concentrations were decreased when the storage periods were increased. p-Cresol was higher at weeks 10 and 14, whereas phenol concentration was higher at weeks 2 and 4; later this trend was reduced until week 14. The skatole concentration was higher at weeks 1 and 2.

### 3.5. Odor Activity Values (OAV) of Slurry and Their Contribution (PR, %)

The OAV measurement is important of the specific method to the detect odor of the sample. It has been calculated as the ratio between the individual component concentration in the sample and the threshold level of this component. If the OAV value is more than 1, it is likely to function as a malodorous component.

**Figure 2:**
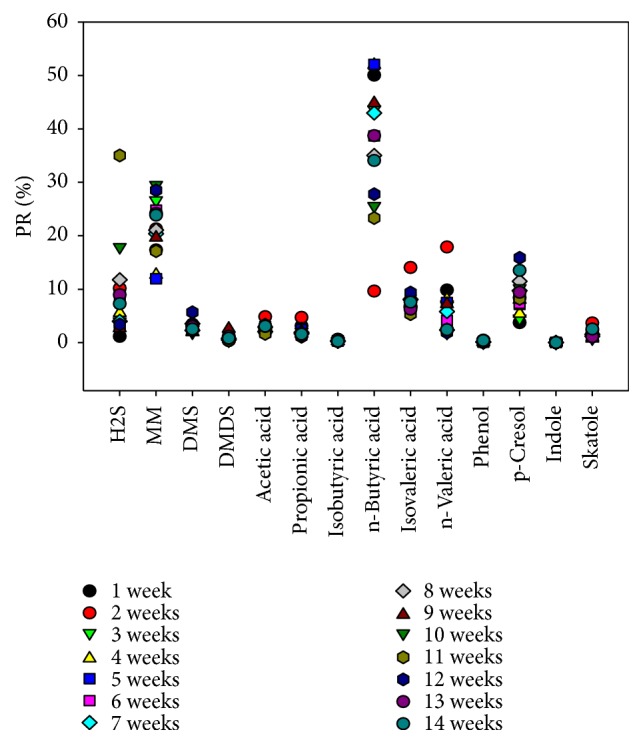
Assessment of odor activity value (OAV) and odor contribution during storage periods.

The average odor contribution of key odorants in each week of the slurry was reflected in Figure 2. Methyl mercaptan and butyric acid were the largest contributor among the designated substances (> 10 to 30 and > 20 to 50%, respectively) to the odor pollution in the slurry at almost every weeks (Figure 2). In addition, p-Cresol is also another contributor for odorizing of slurry ranges from 5% to 20% at the most of the weeks. Particularly, methyl mercaptan and p-Cresol, which are major odor substances, have higher concentration as the storage period is longer. However, the butyric acid level is kept higher for 9 weeks. If the slurry is discharged quickly without storing the slurry for a long period, the concentration of odor substances in the atmosphere can be reduced.

### 3.6. Correlation between Pollutants and Odor Substances Contaminants in Slurry

Phenols among the odor substances in the slurry were strongly correlated with the total pollutants. Phenol production is strongly associated with total nitrogen (81.9%) and ammonium (90.4%). It can be concluded that the increase of nitrogen organic matter in slurry increases the phenols associated malodor (Table 5).

### 3.7. Changes in Bacterial Population in Pig Slurry Based on the Storage Periods

**Figure 3:**
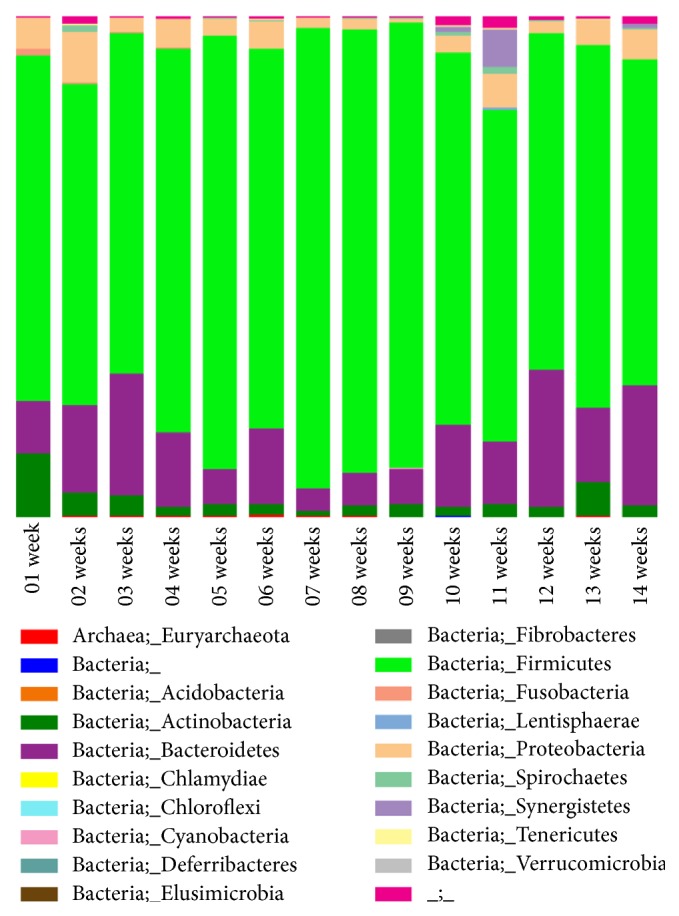
Bacterial taxonomic composition of phylum level in stored pig slurry at different weeks. The sequences were classified according to the EzTaxon-e database with an 80-confidence threshold.

The changes of bacterial populations in the pits of pig slurry were analysed at every week by multiplex bar coded pyrosequencing technique based 16sRNA gene sequences. Taxonomic classification of bacterial communities in the pig slurry was presented in Figure 3. Eighteen phyla were identified throughout storage periods. It indicates that* Firmicute*s,* Bacteroidetes*,* Proteobacteria*, and* Actinobacteria* phylum were dominantly found in the pig slurry. The relative abundances of* Firmicutes* were consistently increased until the 9th week; later this trend was reduced until 14th week.* Bacteroidetes* relative abundances were higher at weeks 3, 12, and 14 than the other storage weeks.* Proteobacteria* relative abundances were fluctuated from the starting to end of the experimental periods.

**Figure 4:**
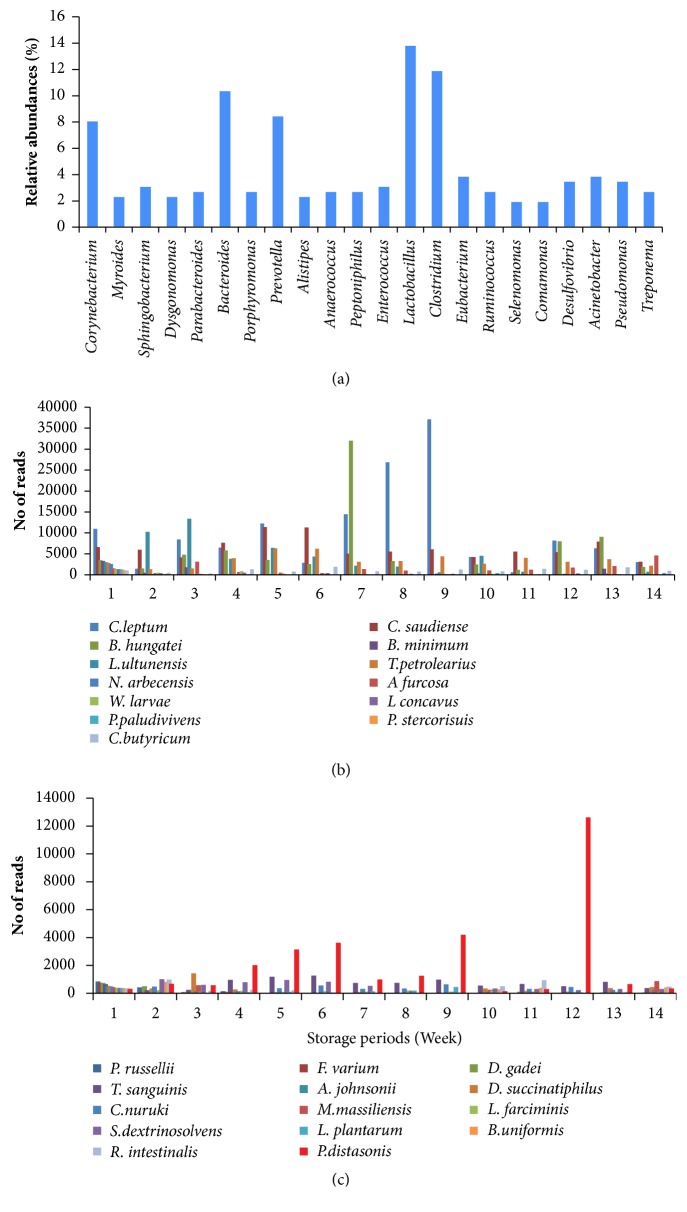
Classification of the dominant bacterial population at genus and species level in slurry samples. (a) Genus level relative abundances of bacterial communities in pig slurry. (b) Species-level dominant bacterial diversity greater than 1000 reads. (c) Species-level dominant bacterial diversity greater than 300 reads.

At the genus level,* Corynebacterium *(8%),* Bacteroides* (10.34%),* Prevotella* (8.4%),* Lactobacillus* (13.79%), and* Clostridium* (11.87%) were dominant in the slurry as compared to another genus (Figure 4(a)). Further, we classified the species level into two categories based on the numbers of reads in all phyla at week 1. First one is bacterial communities greater than 1000 reads and the next one is bacterial communities greater than 300 reads at week 1. In category one, 13 bacterial species showed greater than 1000 reads. Among these,* B. hungatei* and* C. leptum* reads were continuously increased from week 1 to week 7 and week 9, respectively. After that, the bacterial species were drastically reduced until 14th weeks.* P. paludiviens* and C*. saudiense* were increased up to week 3 and week 6, respectively. Most of the bacterial communities were reduced after 3rd week until 14th week (Figure 4(b)). In category two, 14 species demonstrated greater than 300 reads at week 1. Among these,* P. distasonis* drastically increased until week 13. Rests of bacterial species were reduced after 5 to 6 weeks (Figure 4(c)).

### 3.8. Statistical Comparison between Bacterial Population and Odorants in the Slurry during Storage Periods

**Figure 5:**
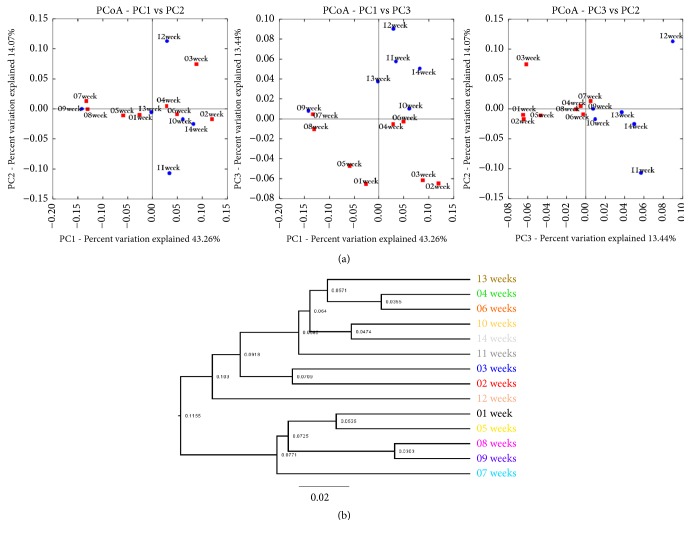
(a) Statistical comparison between bacterial population and odorants in the slurry during storage periods. PCA: principal component analysis; PCoA: principal coordinates analysis. These data were generated by the concentration of odor components and the values of relative abundances in bacterial genus level. (b) Hierarchical clustering results are showing the group by storage periods. Data were constructed by the concentration of odorous compounds and the values of relative abundances in genus level bacterial during different storage periods.

Changes in bacterial communities and concentration of odorants in pig slurry during 14 weeks storage were represented as hierarchical clustering, PCA, and PCoA in Figures 5(a) and 5(b).

## 4. Discussion 

The characterization of sources and reason for the odor emissions are the first criteria to control the odor process. It is the much important way to find the novel ideas for developing a new strategy to control the odorant levels. In this aspect, we investigated the effect of storage periods on key components associated with malodor in deep pit slurry. Furthermore, we analysed correlation between odorants and microbial communities. The medium to long chain volatile organic acids (VOC) were sole reason for the manure odor [24]. Generally, long chain volatile fatty acids (VFA) such as isobutyric, isovaleric, isocaproic acid, and isocaprylic acids have more offensive smells than the short-chain fatty acids [12, 13]. SCFA are the produced during carbohydrates fermentation, but BCFA is produced during protein fermentation. SCFA are the essential source for bacterial growth. It is absorbed and transported into organs and tissues for their energy production [13]. Reduction of SCFA and increases of BCFA indicate a reduction in energy sources availability in pig slurry [25]. In the present study, SCFA and BCFA levels in the pig slurry were continuously increased until 4th and 6th week, respectively. Thereafter, the concentration of SCFA and BCFA was reduced over the 14 weeks of storage periods. However, the level of SCFA was reduced on 14 weeks as compared to the initial level at week 1, whereas BCFA level was higher at weeks 14 as compared with initial level. Similarly, a researcher reported that decreases of SCFAs and increases of BCFAs were noted after 37 days in stored pig slurry [26].

Production of odorants in the slurry is due to incomplete anaerobic decomposition of organic matters especially protein and fermentable carbohydrates [12–14, 27]. Proteins are the major precursors of sulfurous, indolic and phenolic compounds, VFAs, NH3, and volatile amines in the slurry [13, 28]. Phenol, p-Cresol, 4-ethyl phenol, and hydroxylated phenol substituted fatty acids are the main products of tyrosine fermentation. Phenylacetate and phenyl propionates are produced from phenylalanine, but indole and methyl indole are the end products of tryptophan metabolism [29]. Phenols and indoles are produced during bacterial metabolism of tyrosine and tryptophan, respectively, in the stored slurry [13]. It is absorbed through epithelial cells in the large intestine and conjugated with glucuronic acid; furthermore it is converted into glucuronides in the liver by the detoxification process. Then it has been excreted via urine and then hydrolysed to phenols and indoles by fecal *β*-glucuronidase [30]. In this study, maximum phenol and p-Cresol content in the slurry were reached on weeks 6; thereafter, this trend was decreased. However, the content of phenols concentration is always higher in the slurry until week 14 as compared to initial concentration. Indole and skatole concentrations were observed to be maximum in pig slurry from starting to end of the storage periods. Higher production of indoles was noted after 6 weeks of storage. These increases might be due to pH of the pig slurry; it was increased slightly until weeks 14. Many researchers reported that phenol content was increased and indoles were decreased after 8 weeks of storage, which are correlated with pH of the slurry. Generally, phenols were accumulated at low pH, whereas indoles were produced more at high pH [31–34]. Current study demonstrated that pH of pig slurry was slightly changed after 5 weeks throughout storage periods. Lowest pH (5.8) was noted before 5 weeks; thereafter the pH was reached maximum (6.3) at weeks 11.

Finding relationship between bacteria and their roles in slurry is a key factor for finding the mechanism of odorants accumulations. Microbial communities are the main reason behind the production of different types of odorants in slurry using carbohydrates and protein degradations under anaerobic conditions that resulted in the production of organic compounds such as VAFs. Many genera of bacteria have been involved in production of VFAs including* Eubacteria, Peptostreptococcus, Bacteroides, Streptococcus, Escherichia, Megasphaera, Propionibacterium, Lactobacilli,* and* Clostridium*. Among them,* Eubacteria* and* Clostridium* are the potent contributors to the VFAs production [12]. Therefore, identification of bacterial genera in pig manure is an important way to control the odorants productions. Numbers of researchers investigated bacterial community in pig slurry using culture methods [35–37]. Nowadays, multiplex bar coded pyrosequencing methods based on 16rRNA genes are used to identify the bacterial communities [18, 38, 39]. In the present study,* Firmicute*s,* Bacteroidetes*,* Proteobacteria*, and* Actinobacteria* phylum were dominantly found the pig slurry. Particularly* Firmicute*s phylum was consistently increased until weeks 9; thereafter, the trend was changed. However,* Bacteroidetes* phylum was reduced at mid storage periods; later and before, this phylum increased.* Proteobacteria* and* Actinobacteria* phylum were continuously reduced after second weeks. At genus level,* Corynebacterium, Bacteroides, Prevotella, Lactobacillus*, and* Clostridium* were dominantly found in the pig slurry. At species level*, B. hungatei*,* C. leptum*, and* P. distasonis* were highly found in pig slurry at weeks 7, 9, and 14, respectively. Differences in bacterial groups dominant might be due to environmental changes in pig slurry in pits during storage periods. These bacteria can use the amino acids as energy for their growth in stored manure [27]. PCA and PCoA score plots support our current study; it is shown based on the bacterial groups, and the odorants level was changed in pig slurry. The bacterial composition in slurry is correlated with the key odorants production.

Concentrations of odorous compounds produced in pig slurry were correlated with bacterial communities. Phenols, indoles, BCFA, and SCFA productions were strongly correlated (> 60%) with* Firmicute*s,* Proteobacteria, Bacteroidetes, Actinobacteria*, and* Euryarchaeota* (Supplement Figure 1). These phyla were dominantly found in the deep pig slurry. Cotta et al., 2003, and Spoelstra et al., 1978, previously isolated and identified the* Firmicute*s, and* Bacteroides* from stored animal manure. These bacteria can able to produces different types of enzymes which degrade the wide range of carbohydrates and proteins; especially *β*-glucuronidase from bacteria can have the ability to hydrolyse the glucuronides to phenols and indoles [40]. In addition, hydrophobic molecules such as phenols, indoles, and their precursor can effectively transfer to the bacterial cell wall of* Bacteroides* [33, 41].

*Methanosphaera, Bifidobacterium, Chryseobacterium, Tissierella, Enterococcus, Anaerofilum, Fastidiosipila, Veillonella, Oligella, Comamonas, Campylobacter, Globicatella, Pseudomonas, Stenotrophomonas, Succinivibrio, Klebsiella, Bacteroides, *and* Porphyromonas* genus were positively correlated (> 60%) with phenols, indoles, acetic acid, butyric acid, isobutyric acid, and isovaleric acid productions. These genera are the phyla of* Firmicutes, Proteobacteria, Bacteroidetes, Actinobacteria*, and* Euryarchaeota*. Our current study has differed from the previous investigation; they have stated that the phylum of Tenericutes and Cloacamonas is positively correlated with odor compounds production [21]. The current study clearly stated that new phylum of bacteria is also involved in key odor components production in pig slurry during storage periods. Generally, key compounds associated with the offensive odor such SFCA, phenols, indoles, H_2_S, MM, DMS VOCs, CO_2_, and H_2_ were produced in pig slurry during fermentation and degradation of organic matters by different bacterial genera, such* Eubacteria, Bacteroides, Streptococcus, Megasphaera, Lactobacilli*, and* Clostridium* (Roderick et al., 1998).* Bacteroides, Lactobacilli, Clostridium*, and* Bifidobacterium* play key role in the production of phenols and indoles from tyrosine, phenylalanine, and tryptophan [29, 32].

## 5. Conclusion 

The data from current study demonstrated that key odor components and microbial diversity in the stored pig slurry in deep pits were affected during different storage periods. The levels of odorants and bacterial flora in pig slurry were altered every week. BCFA, phenols, and indoles concentrations were higher at 14 weeks as compared to the initial concentration whereas the final concentration of SCFA was reduced in week 14 as compared to the initial concentration of SCFA. Most of the physiochemical factors are being fluctuated at different storage periods. However, COD, BOD, and SS level were lowered at the end of the storage periods. The atmospheric concentration of H_2_S was increased whereas the concentration of MM was reduced at weeks 14 compared to the initial concentration at week 1. Microbial ecology suggested that* Firmicutes* and* Bacteroidetes* were dominantly found in pig slurry throughout storage periods. Furthermore, key compounds production is associated with offensive odors in pig slurry which were correlated with bacterial communities. Phenols, indoles, SCFA, and BCFA productions were positively correlated with* Firmicutes *and* Bacteroidetes, *followed by* Proteobacteria, Actinobacteria, *and* Euryarchaeota *phyla. It seems that bacterial species under* Firmicutes* and* Bacteroidetes *phyla play an important role in the offensive odor compounds production via protein and carbohydrate degradations. Taken together, the prevention of these phyla bacterial growth and early removal of pig slurry from deep pits might reduce the key odor compound production from pig farming house.

## Figures and Tables

**Table 1:** Environmental condition in pig farming house during different storage periods.

Weeks	Temperature(°C)	Humidity (%)	No. of pigs in fattening stage (832*∗*1195m^2^)
1	27.7	85	93
2	27	85	86
3	28	82	87
4	27	82	85
5	28	89	90
6	29	86	98
7	28.5	86	95
8	29	86	92
9	28.3	86	89
10	28.7	87	91
11	28	86	85
12	28.5	87	83
13	27.5	81	98
14	28.2	81	79

**Table 2:** Key odor components in pig slurry during different storage periods.

Parameters (ppm)	1	2	3	4	5	6	7	8	9	10	11	13	14
SCFA^1^	2234±137	3874±402	5403±155	8132±50	6637±64	7771±121	5808±173	3620±25	2976±32	2670±146	2069±107	2561±65	1934±43
ACA	1033±63	1899±127	2270±18	3872±30	3190±30	3917±58	2629±71	1898±9	1594±24	1270±71	956±49	1261±30	949±23
PPA	412±25	715±74	793±7	1162±7	641±5	968±15	770±24	352±2	149±3	434±22	334±17	339±8	280±3
BTA	612±37	1137±118	1549±16	2481±16	2110±20	2263±38	1663±51	1071±10	992±1	745±42	607±30	793±20	537±15
VLA	176±10	278±28	788±113	616±3	695±7	622±10	744±3	298±3	241±2	220±10	172±9	212±5	166±8
BCFA^2^	96±5	265±27	269±5	524±5	188±2	667±11	374±16	273±3	101±3	143±3	177±10	209±5	127±4
IBA	37±2	101±11	100±2	200±3	74±0.8	247±4	133±6	100±1.	40±2	49±0.9	62±3	74±2	46±0.7
IVA	58±3	164±16	169±3	323±2	113±1	420±6	240±10	173±2	61±1	93±3	114±6	135±3	81±4
Phenols^3^	69±1.6	141±3	134±1	191±2	152±0.1	220±2	194±2	180±1	111±1	110±0.2	135±0.4	175±0.8	137±0.8
PhAl	7±0.3	15±0.2	13±0.0	21±0.4	17±0.0	19±0.2	17±0.3	18±0.2	14±0.1	11±0.0	12±0.0	15±0.2	11±0.1
P-C	62±1	126±3	121±1	170±17	135±0.1	200±2	176±2	162±1	96±1	99±0.2	123±0.5	160±0.6	125±0.7
Indoles ^4^	11±0.0	13±0.1	19±0.1	19±0.1	24±0.0	27±0.3	29±0.4	24±0.2	34±0.4	23±0.6	25±0.1	32±0.2	24±0.3
ID	5±0.0	6±0.1	12±0.1	6±0.1	14±0.0	13±0.1	16±0.2	13±0.1	17±0.2	7±0.1	9±0.0	12±0.1	8±0.1
SK	6±0.0	7±0.0	7±0.0	13±0.1	9±0.0	13±0.2	12±0.2	10±0.1	17±0.2	15±0.5	16±0.1	19±0.1	15±0.2

^1)^ SCFA (short chain fatty acids) = acetic acid (ACA) + propionic acid (PPA) + butyric acid (BTA) + valeric acid (VLA).

^2)^ BCFA (branch chain fatty acids) = isobutyric acid (IBA) + isovaleric acid (IVA).

^3)^ Phenols = phenol + P-Cresol (P-C).

^4)^ Indoles = indole (ID) + skatole (SK).

**Table 3:** Physiochemical analysis of pig slurry during different storage periods.

Parameters	1	2	3	4	5	6	7	8	9	10	11	13	14
BOD (mg/L)	37020	57060	56220	79740	86940	75780	73980	74340	99540	41460	29580	55260	33060
COD (mg/L)	13600	18400	21600	27200	31200	32000	22400	24000	32800	19200	10400	16000	12000
SS (mg/L)	13400	26500	22100	62700	76307.7	65301.2	29700	39500	64800	37200	7500	21600	8300
P_2_O_5_ (%)	0.145	0.260	0.235	0.330	0.440	0.400	0.280	0.215	0.325	0.135	0.100	0.235	0.135
TKN (%)	0.3	0.5	0.5	0.7	0.8	0.7	0.6	0.6	0.7	0.4	0.3	0.5	0.3
NH _4_ -N(%)	0.1231	0.2348	0.2159	0.3293	0.3428	0.3371	0.2979	0.2414	0.3126	0.1420	0.1563	0.2371	0.1536
NO _3_ -N(%)	0.00215	0.00210	0.00246	0.00187	0.00187	0.00186	0.00196	0.00215	0.00211	0.00208	0.00256	0.00212	0.00205
pH	5.8	5.9	5.8	6.0	5.9	6.0	6.0	6.0	6.0	6.0	6.3	6.0	6.0
Moisture (%)	95.90	94.42	94.59	91.35	88.31	89.36	93.57	93.60	89.27	94.57	97.47	92.73	97.30
OM (%)	2.733	3.983	3.930	6.499	9.557	8.785	4.627	4.764	8.815	3.998	1.401	5.653	1.545

BOD: biological oxygen demand; COD: biological chemical demand; SS: solid suspension; P_2_O_5_: phosphorus pentoxide; TKN: total Kjeldahl nitrogen; NH _4_ –N: total ammonium; NO _3_ –N: total nitrate, OM: organic matter.

**Table 4:** Concentration of odor components at an atmospheric air during different storage periods.

Week		1	2	3	4	5	6	7	8	9	10	11	13	14
Complex odorants (OU)		10,000	14,422	3,000	6,694	14,422	20,800	10,000	4,481	3,000	4,481	10,000	14,422	20,800

Sulfur Compound	H2S	62	297	213	286	145	353	170	334	132	754	1,685	276	227
	MM	186	124	286	131	105	197	173	119	190	249	165	149	149
	DMS	32.4	20.0	20.4	20.0	21.9	20.5	19.4	20.1	19.9	21.6	31.3	18.6	15.8
	CS2	3.38	3.58	4.19	3.70	3.68	4.24	3.86	3.69	4.11	4.13	7.27	4.39	4.06
	DMDS	12.0	13.5	18.6	21.8	30.7	17.7	19.9	8.98	75.3	10.3	11.1	22.3	15.4

VOCs (ppb)	MEK	48.9	59.6	38.0	44.9	39.4	41.6	33.7	42.0	76.9	36.1	37.5	57.1	67.7
	Benzene	0.41	0.41	0.41	0.41	0.41	0.41	0.82	0.41	0.41	0.41	0.41	0.41	0.41
	MIBK	5.04	3.71	0.34	0.34	3.38	3.43	0.34	0.34	2.89	3.94	3.35	3.11	3.12
	Toluene	153	279	112	175	178	211	7.14	33.4	159	278	96.7	12.9	216
	n-Butyl acetate	5.13	5.65	11.0	5.27	0.71	0.71	4.52	4.27	5.66	5.80	0.00	0.71	5.26
	Isobutyl alcohol	10.9	4.45	0.37	0.71	8.61	8.12	7.69	6.73	10.4	7.60	10.5	9.9	11.9
	Ethylbenzene	2.82	2.29	3.14	2.78	2.01	2.80	0.38	0.43	2.68	3.72	1.54	0.43	2.32
	m-Xylene	2.36	2.47	3.02	2.52	2.27	2.67	1.68	1.65	2.63	2.91	2.25	2.51	2.49
	p-Xylene	3.04	3.32	5.46	3.47	2.79	3.70	1.48	1.42	3.58	4.42	2.63	3.47	3.28
	o-Xylene	22.1	24.0	42.0	22.3	14.5	24.2	13.9	13.6	22.7	27.7	20.7	21.9	24.3
	Styrene	4.59	4.87	4.35	4.25	4.65	4.61	3.69	3.53	4.35	3.46	5.27	4.61	5.02

VFA s(ppb)	Acetic acid	1,484	1,619	1,231	1,363	1,109	876	*1,002*	941	1,573	1,164	895	1,065	1,092
	Propionic acid	671	552	433	515	431	271	324	189	328	247	210	163	198
	Isobutyric acid	48.3	54.3	44.0	50.0	46.6	34.4	39.2	24.0	39.9	28.8	26.7	19.6	24.4
	n-Butyric acid	376	394	328	374	320	215	256	139	304	151	157	167	149
	Isovaleric acid	35.8	41.0	34.2	37.8	35.3	28.0	34.4	22.8	34.9	25.6	25.6	19.3	23.7
	n-Valeric acid	106	105	76.8	83.4	66.1	33.0	49.5	13.4	69.8	15.5	21.4	14.9	15.1

Phenols (ppb)	Phenol	1.84	7.55	1.61	2.82	2.80	2.05	3.09	1.83	3.53	4.15	7.35	1.85	6.77
	p-Cresol	21.7	26.5	26.2	30.1	33.9	31.6	44.6	35.1	41.9	46.3	42.6	31.4	45.6

Indoles (ppb)	Indole	0.40	0.40	0.47	0.40	0.43	0.53	0.40	0.40	0.40	0.40	0.40	0.40	0.52
	*Skatole*	1.11	1.20	0.49	0.59	0.57	0.74	0.73	0.38	0.41	0.81	1.05	0.38	0.89

H_2_S: hydrogen sulfide; MM: methyl mercaptan; DMS: dimethyl sulfide; CS2: carbon disulfide; DMDS: dimethyl disulfide; MEK: methyl ethyl ketone; MIBK: methyl isobutyl ketone.

**Table 5:** Correlation between key odorants generations and physiochemical parameters.

	PhAl	P-C	ACA	IBA	BTA	IVA	SCFA	BCFA	Phenols
BOD (mg/L)	*0.732*	0.432	0.472	0.370	0.457	0.348	0.442	0.357	0.464

COD (mg/L)	0.659	0.432	0.548	0.448	0.498	0.425	0.497	0.433	0.457

SS (mg/L)	0.676	0.379	0.583	0.434	0.552	0.403	0.533	0.415	0.409

P_2_O_5_(%)	*0.741*	0.508	0.668	0.524	0.666	0.493	0.647	0.505	0.535

TKN(%)	**0.819**	0.572	0.641	0.545	0.625	0.523	0.609	0.531	0.600

NH_4_-N(%)	***0.904***	*0.765*	0.658	0.643	0.633	0.633	0.623	0.637	*0.785*

Moisture (%)	-0.670	-0.412	-0.551	-0.422	-0.529	-0.399	-0.509	-0.408	-0.440

OM (%)	0.651	0.397	0.528	0.403	0.505	0.381	0.485	0.389	0.424

Underline numbers: 60% positive correlation (p<0.05).

Italic numbers: 70% positive correlation (p<0.05).

Bold numbers: 80% positive correlation (p<0.05).

Bold italic numbers: 90% positive correlation (p<0.05).

## Data Availability

The data used to support the findings of this study are available from the corresponding author upon request.

## Supplementary Materials

Supplementary Materials Correlation study between the microbial populations and odorous substances in pig slurry. The correlation between bacteria at genus level (*Bifidobacterium*,* Bacteroides*,* Porphyromonas*,* Chryseobacterium*,* Tissierella*,* Globicatella*,* Enterococcus*,* Anerofilum*,* Fastidiosipila*,* Veillonella*,* Oligella*,* Comamonas*,* Campylobacter*,* Klebsiella*,* Succinivibrio*,* Stenotrophomonas*, and* Pseudomonas*) and odorous substances (phenols, indoles, VFAs, and VOCs) in the slurry were performed using the SAS 9.2 software. Data with a p-value of 0.05 or less and R2 values greater than 0.6 were selected and displayed as scatter diagrams. The R2 value of the calibration curve of VFAs was taken between 0.996 ~ 0.999, and the R2 value of phenols and indoles was taken between 0.991 ~ 0.993. The scatter plot confirms various microorganisms strongly correlated (p <0.05) with odorous substances (> 60%). Among the methanosphaera a positive correlation with volatile fatty acids (VFAs) such as acetic acid (ACA), butyric acid (BTA), isobutyric acid (IBA), + isovaleric acid (IVA), and valeric acid (VLA) was shown. In the case of Veillonella, phenolics such as PhAl and indole (ID) were correlated similarly with volatile organic acids (VOCs). Although it does not reveal the exact mechanism of the microorganisms, it can be assumed that the microorganisms have a positive correlation for producing odorous substances as byproducts during metabolism, and the microorganisms having a negative correlation are likely to ingest odorous substances as nutrients. Overall data suggest that it is an important way to identify the relationship between odorous substances and the microbial mechanisms in the slurry in pigs.
