# Supplementary material for: Biochemical Changes and Biological Origin of Key Odor Compound Generations in Pig Slurry during Indoor Storage Periods: A Pyrosequencing Approach
Source: Biomed Res Int. 2018 Sep 12;2018:3503658. doi: 10.1155/2018/3503658 (PMC6157135; doi:10.1155/2018/3503658)
Supplement: Supplementary Materials — Correlation study between the microbial populations and odorous substances in pig slurry. The correlation between bacteria at genus level (Bifidobacterium, Bacteroides, Porphyromonas, Chryseobacterium, Tissierella, Globicatella, Enterococcus, Anerofilum, Fastidiosipila, Veillonella, Oligella, Comamonas, Campylobacter, Klebsiella, Succinivibrio, Stenotrophomonas, and Pseudomonas) and odorous substances (phenols, indoles, VFAs, and VOCs) in the slurry were performed using the SAS 9.2 software. Data with a p-value of 0.05 or less and R2 values greater than 0.6 were selected and displayed as scatter diagrams. The R2 value of the calibration curve of VFAs was taken between 0.996 ~ 0.999, and the R2 value of phenols and indoles was taken between 0.991 ~ 0.993. The scatter plot confirms various microorganisms strongly correlated (p <0.05) with odorous substances (> 60%). Among the methanosphaera a positive correlation with volatile fatty acids (VFAs) such as acetic acid (ACA), butyric acid (BTA), isobutyric acid (IBA), + isovaleric acid (IVA), and valeric acid (VLA) was shown. In the case of Veillonella, phenolics such as PhAl and indole (ID) were correlated similarly with volatile organic acids (VOCs). Although it does not reveal the exact mechanism of the microorganisms, it can be assumed that the microorganisms have a positive correlation for producing odorous substances as byproducts during metabolism, and the microorganisms having a negative correlation are likely to ingest odorous substances as nutrients. Overall data suggest that it is an important way to identify the relationship between odorous substances and the microbial mechanisms in the slurry in pigs. [file 3503658.f1.docx]

**S1- A scatter plot for odor substances with a correlation between microbial and odorous substances greater than 60%**

|  |  |
| --- | --- |
|  |  |
|  |  |
|  |  |
|  |  |

|  |  |
| --- | --- |

|  |  |
| --- | --- |

|  |  |
| --- | --- |

|  |  |
| --- | --- |
|  |  |

|  |  |
| --- | --- |
|  |  |

|  |  |
| --- | --- |

|  |  |
| --- | --- |
|  |  |
|  |  |

|  |  |
| --- | --- |
|  |  |

|  |  |
| --- | --- |
|  |  |
|  |  |
|  |  |

|  |  |
| --- | --- |

|  |  |
| --- | --- |
|  |  |
|  |  |

|  |  |
| --- | --- |

|  |  |
| --- | --- |
|  |  |
